# Evaluating the Toxicity and Histological Effects of Al_2_O_3_ Nanoparticles on Bone Tissue in Animal Model: A Case-Control Study

**DOI:** 10.1155/2020/8870530

**Published:** 2020-11-22

**Authors:** Hossein Soltaninejad, Hadi Zare-Zardini, Amir Ali Hamidieh, Mohammad Reza Sobhan, Seyed Houssein Saeed-Banadaky, Mohammad Amir Amirkhani, Behnaz Tolueinia, Mohsen Mehregan, Mahnaz Mirakhor, Farzaneh sadat Eshaghi

**Affiliations:** ^1^Department of Nano Biotechnology, Faculty of Biological Sciences, Tarbiat Modares University, Tehran 14115, Iran; ^2^Iranian Tissue Bank & Research Center, Tehran University of Medical Sciences, Tehran, Iran; ^3^Hematology and Oncology Research Center, Shahid Sadoughi University of Medical Sciences, Yazd, Iran; ^4^Medical Nanotechnology & Tissue Engineering Research Center, Yazd Reproductive Sciences Institute, Shahid Sadoughi University of Medical Sciences, Yazd, Iran; ^5^Department of Sciences, Farhangian University, Isfahan, Iran; ^6^Pediatric Cell Therapy Research Center, Tehran University of Medical Sciences, Tehran, Iran; ^7^Department of Orthopedics, Shahid Sadoughi University of Medical Sciences, Yazd, Iran; ^8^Stem Cell and Regenerative Medicine Institute, Tehran University of Medical Sciences, Tehran, Iran; ^9^University of Applied Science and Technology of Sistan and Baluchestan, Minushargh Branch, Zahedan, Iran; ^10^Department of Physics, Payam-e-noor University, Zahedan, Iran; ^11^Shahid Sadoughi Hospital, Shahid Sadoughi University of Medical Sciences, Yazd, Iran; ^12^Department of Genetics, Faculty of Medicine, Shahid Sadoughi University of Medical Sciences, Yazd, Iran

## Abstract

The applications of nanostructures have been limited by their different toxicities. So, the investigation of these toxicities is necessary before nanostructure application. This study aimed to evaluate the effect of aluminum oxide (Al_2_O_3_) nanoparticles on bone density in Wistar rat. Al_2_O_3_ nanoparticle was prepared by the sol-gel method. Characterization was done by X-ray diffraction (XRD) and transmission electron microscopy (TEM). Sixty-four male adult Wistar rats were divided into eight groups including six groups intravenously treated with Al_2_O_3_ nanoparticle at concentrations of 25, 50, 100, 250, 500, and 1000 *µ*g/ml: one group received food and water as the control group, and one group received food and water as well as intravenously distilled water as an injection control group. After 41 days, bone density was analyzed by dual-energy X-ray absorptiometry (DEXA). According to X-ray diffraction, the average particle size for Al_2_O_3_ nanoparticles was 20.85 nm. The data of densitometry showed that the bone density of right and left foot was reduced in concentrations of 250, 500, and 1000 *µ*g/ml that were statistically significant in comparison with the control group. The reduction of bone density was increased with the enhancement of nanostructures concentration. The effect of Al_2_O_3_ nanoparticles on bone density was similar in the left and right legs. Histopatholological assessment also showed that Al_2_O_3_ nanoparticles (250, 500, and 1000 *µ*g/ml) lead to significant reduction of trabeculae. Empty lacunae are observed in these three groups. Considering that high concentrations of Al_2_O_3_ nanoparticles had toxicity on bone tissue, it must be used by more caution, especially its use as a coating in different devices such as implants, surgical instruments, and bone prostheses.

## 1. Introduction

Recently, nanostructures have found increased applications in technology, research, and medicine [1–3]. These compounds have unique properties because of their small size. In the past decades, the use of nanotechnology has risen in science due to their wide range of biomedical applications, for example, in antimicrobial agents [3], coating [4], drug delivery [5, 6], medical imaging [7], the optoelectronic device [8], suitable catalyst [9], and other beneficial applications [10–15]. Besides beneficial properties, nanostructures have dangerous toxicity on all of the worlds, especially on human life [16, 17]. For some types of particles, the smaller size leads to a greater surface area/volume ratio and higher chemical reactivity and biological activity [18, 19]. The toxicity of different nanostructures has been proven on different organs such as blood, lungs, liver, skin, gut, heart, reproductive organ, and other organs [20]. Once in the bloodstream, nanostructures can be transported around the body and be taken up by organs and tissues, including the brain, heart, liver, kidneys, spleen, bone marrow, and nervous system [21]. Nanomaterials are toxic for human tissue and cell cultures, resulting in increased oxidative stress, inflammatory cytokine production, and cell death. Nanostructures may be taken up by cell mitochondria and the cell nucleus. Nanomaterials can cause DNA mutation and induce major structural damage to mitochondria. The one organ that effects of nanostructures is bone. Nanostructures can penetrate deeper into skin layers and possibly be absorbed into the systemic circulation and accumulate in tissues, especially in bone because of the development of nanodevices that are used in surgical instruments and bone prostheses. So, the evaluation of the toxicity of nanostructures on bone is a very important field of nanotoxicology. Alumina is one of the inert biomaterials used in implants [22, 23]. It is, therefore, a biodegradable material, well-tolerated by the biological environment. This nanostructure has been defined as a suitable compound that is used in different fields of life. Thus, the present paper aims to investigate the toxic effect of Al_2_O_3_ on the bone.

## 2. Materials and Methods

### 2.1. Materials

Aluminum chloride (AlCl_3_) and ethanol were purchased from Sigma. Other used chemicals were analytical grade.

#### 2.1.1. Synthesis and Characterizations of Nanostructures

According to our previous study [24] and other related articles [25–27], the sol-gel method was used for the synthesis of nanostructures. In brief, AlCl_3_ ethanolic solution (0.1 M) was prepared. NH_3_ solution (28%) was added to AlCl_3_ ethanolic solution. In this stage, a gel was formed. This acquired gel was incubated at room temperature for 30 hours. Then, the acquired agent was dried at 100°C for 24 hours.

X-ray diffraction (XRD) and transmission electron microscope (TEM) were used for identifying the crystalline mineralogical phases of the powders and micrographs, respectively.

The size of nanocrystals has been calculated using the Debye–Scherrer formula using reflection from the XRD pattern. Debye–Scherrer formula for crystallite size determination is given as follows:

(1) $$\begin{array}{c} D=\frac{0.94\lambda }{\beta \cos\theta }, \end{array}$$

where, *D* is the crystallite size, *λ* is the wavelength of X-ray, *β* is the full width at half maximum, and *θ* is Bragg's angle.

#### 2.1.2. Investigation of Bone Density

For investigating the effect of Al_2_O_3_ nanoparticles on bone density, 64 male adult Wistar rats (mean body weight = 245 g) were used. All ethics were well-considered standards in compliance with the Animal Welfare Act. The research was approved by the ethics committee of Shahid Sadoughi University of Medical Sciences. The Wistar rats were kept in metal cages with standard conditions (environment temperature: 25°C, suitable humidity: 50%, and sufficient light: 12/12 hours light/dark). A proper diet consisting of fats, carbohydrates, protein, and vitamins was given. Rats were randomly divided into 8 groups (8 rats in each group) which are as follows:

  The control group (group 1): regular foods
  Injection control (group 2): intravenous injection of 0.5 ml distilled water alongside regular food
  Six treatment groups: intravenous injection of 0.5 ml Al_2_O_3_ nanoparticles in different concentrations alongside regular food as group 3 (25 *µ*g/ml), group 4 (50 *µ*g/ml), group 5 (100 *µ*g/ml), group 6 (250 *µ*g/ml), group 7 (500 *µ*g/ml), and group 8 (1000 *µ*g/ml)

Following the treatment of eight Wistar rats in each group during 40 days, dual-energy X-ray absorptiometry (DEXA) was used for bone densitometry. In brief, in this method, two sources of the X-ray were irradiated to bone. In a higher density of bone, the X-ray absorption was greater. This condition leads to less radiation reaching the detector. The radiation received to the detector was calculated and converted to the scale of bone density as g/cm^2^ by computer analysis.

#### 2.1.3. Histological Examination

For histological evaluation, bone samples (control group and group 8) were prepared and fixed in paraformaldehyde. After 24 hours, samples were dehydrated by ethanol and embedded in paraffin. Thin slices were prepared by block cutting. Staining was done with hematoxylin and eosin (H&E) solution. Qualitative assessment was done by a pathologist under a light microscope based on experiences and references.

#### 2.1.4. Statistical Analysis

The data were analyzed by SPSS (version 20). Means ± SD were calculated for the average bone density. These calculated data were compared between groups by one-way analysis of variance (ANOVA) followed by Tukey's test as post hoc. A *p* value of less than 0.05 was considered significant.

## 3. Results

### 3.1. Synthesis and Characterizations of Nanostructure

**Figure 1:**
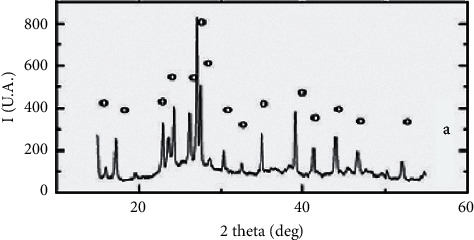
The XRD pattern of alumina powder obtained by sol-gel method from aluminum chloride (AlCl3), dried 100^o^C/24 h.

**Figure 2:**
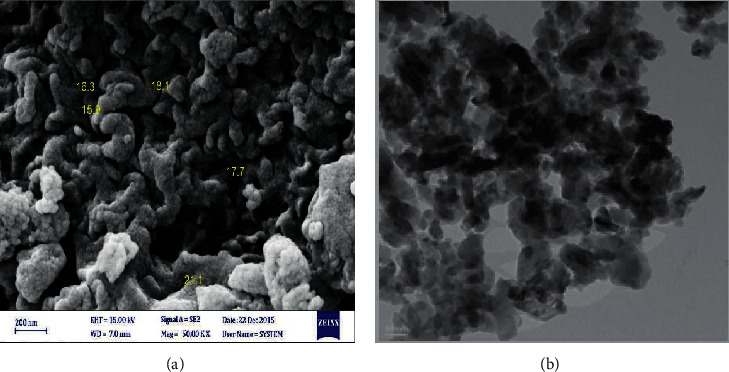
SEM (a) and TEM (b) images of alumina powder obtained by sol-gel method.

Al_2_O_3_ nanoparticles were synthesized by the sol-gel method and characterized by X-ray diffraction and scanning electron microscopy (TEM).  Figure 1 shows the XRD pattern of alumina powder. The particle size of Al_2_O_3_ nanoparticles was 20.85 nm.  Figure 2 shows the transmission electron microscopy (TEM) micrographs of alumina powder obtained from aluminum chloride solution (0.1 M), heat-treated at 100°C for 24 hours. There are two types of particles with different geometries, namely, needle-shaped particles with an average particle size below 30 nm and spherical particles with an average size below 20 nm. This figure also showed the SEM micrographs of synthesized nanostructures and proper synthetic processes.

### 3.2. Bone Evaluation

**Figure 3:**
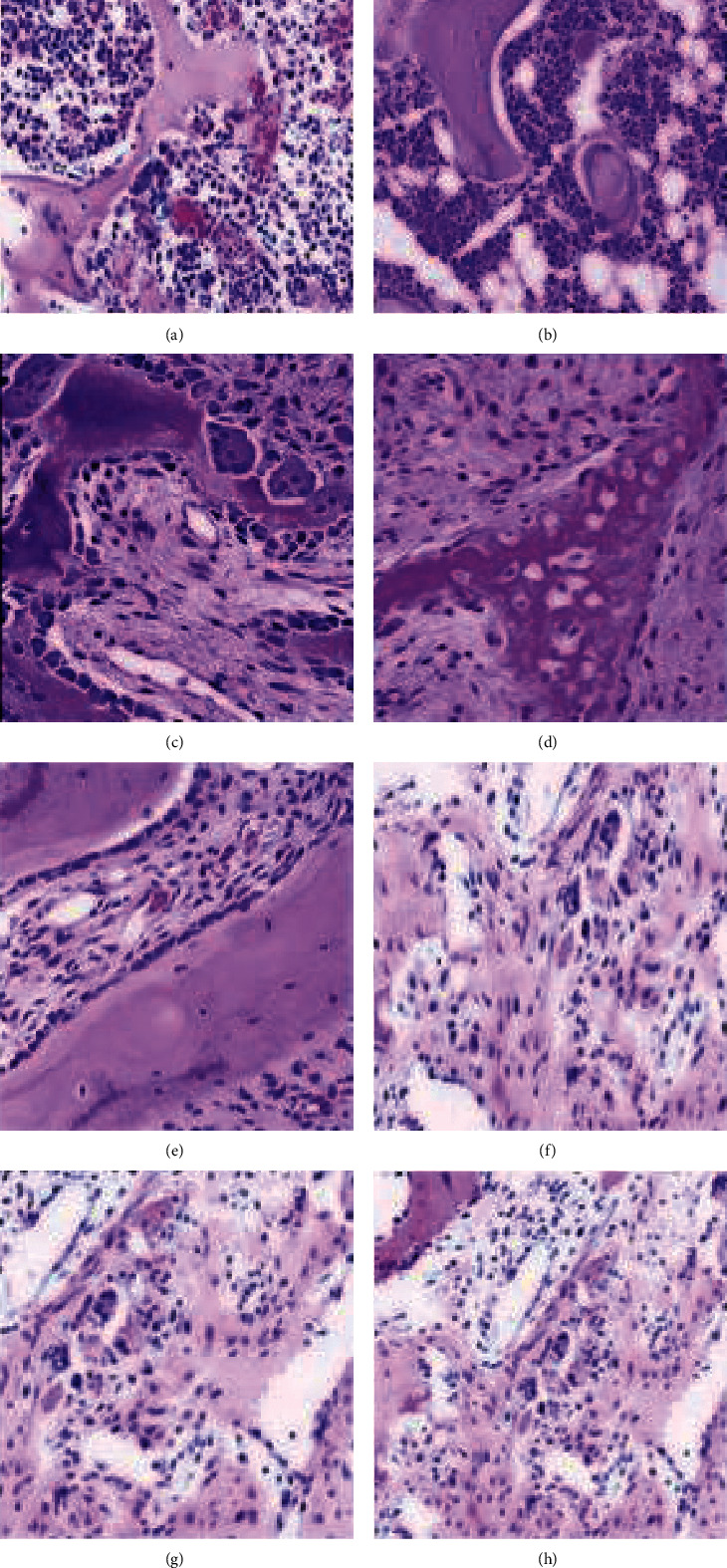
Comparison of bone histology of control (a), injection control (b), treated with 0.5 ml Al_2_O_3_ nanoparticles (25 *µ*g/ml) (c), treated with 0.5 ml Al_2_O_3_ nanoparticles (50 *µ*g/ml) (d), treated with 0.5 ml Al_2_O_3_ nanoparticles (100 *µ*g/ml) (e), treated with 0.5 ml Al_2_O_3_ nanoparticles (250 *µ*g/ml) (f), treated with 0.5 ml Al_2_O_3_ nanoparticles (500 *µ*g/ml) (g), and treated with 0.5 ml Al_2_O_3_ nanoparticles (1000 *µ*g/ml) (h) by H&E staining.

Bone densitometry was done on both left and right feet. Being treated with different concentrations of Al_2_O_3_ nanoparticle led to change in the bone density in all treated groups. Investigating the mean of bone density in different groups showed the highest density at the lowest concentration of nanostructure (25 *µ*g/ml, 0.1498 ± 0.001 and 0.1485 ± 0.002 g/cm^2^ for left and right foot, respectively) and the lowest bone density at the highest concentration (1000 *µ*g/ml, 0.1012 ± 0.002 and 0.098 ± 0.003 g/cm^2^ for left and right foot, respectively) (Table 1). Based on the ANOVA test, the differences between some of the means are statistically significant. The results of Tukey's test showed that the change of bone density in groups treated with concentrations of 250, 500, and 1000 *µ*g/ml of Al_2_O_3_ nanoparticle had a significant reduction compared to the control group (*p* < 0.05). The observed reductions in the treatment group treated at the concentration of 25, 50, and 100 *µ*g/ml were not significant compared to the control group (*p* > 0.05). These changes were observed in both feet. Histopathological assessment showed that 1000 *µ*g/ml of Al_2_O_3_ nanoparticles led to significant reduction of trabeculae. Empty lacunae are observed in this group. In treated groups with 500 *µ*g/ml, empty lacunae and reduction of trabeculae are also observed. In treated groups with 250 *µ*g/ml, the side effect of nanoparticle is less than treated groups with 500 and 1000 *µ*g/ml. However, reduction of trabeculae was also observed in this group, to some extent. The bone matrix is reduced in treated groups with 250, 500, and 1000 *µ*g/ml of nanoparticle, and osteocyte cell destruction is evident. The rate of destruction of the bone matrix and blades is higher in treated groups with 1000 *µ*g/ml than other groups. In control group, intact and dense bone with complete trabecular structure was observed. The similar condition was observed in the injection control group and treated groups with 25, 50, and 100 *µ*g/ml of Al_2_O_3_ nanoparticles. The overall appearance of the bones in control group, injection control group, and treated groups with 25, 50 and 100 *µ*g/ml of Al_2_O_3_ nanoparticles was normal. A healthy bone matrix with cylindrical osteocyte and lacunar around it is clear in these groups (Figure 3).

## 4. Discussion

Nanomaterials show toxicological properties in comparison with the same substance in the bulk form [28]. The use and release of nanostructures into the environment can affect each stage of a life cycle [29]. The knowledge about the toxicity of nanomaterials is incomprehensive, especially long-term environmental and chronic health impacts [30]. These nanomaterials can lead to a defect in all organs of the body such as liver, blood, brain, lung, and, even, bone tissue [31–34]. In this study, the effect of Al_2_O_3_ nanoparticles on bone density was investigated by DEXA method in Wistar rats. In X-ray analysis of synthesized Al_2_O_3_ nanoparticles, Shimadzu diffractometer XRD 6000-Ni-filtered CuK*α* (*λ* = 1.5406 Å) radiation, scanning speed of 0.02°/min, in 2*θ* = 10–70 deg. range was used [24]. Based on this analysis, the particle size of Al_2_O_3_ nanoparticles was 20.85 nm. Based on these results and similar studies [35, 36], we synthesized relatively highly pure nanostructures without any unwanted impurities. In TEM graph, needle- and sphere-shaped nanoparticles were seen. Similar to XRD data, the mean size of synthesized Al_2_O_3_ nanoparticles was below 30 nm. In TEM graph, agglomeration of Al_2_O_3_ nanoparticles was also seen. SEM analysis was confirmed in TEM and XRD data. Surface charge is one of the important reasons for the stability of colloidal particles, especially metal oxide nanoparticles. The surface charge of nanoparticles is pH-dependent and raises the intrinsic properties of the oxides on their surface. Observed agglomeration could be due to this fact [25, 26, 36]. The results showed that the treatment of Wistar rats with different concentrations of Al_2_O_3_ nanoparticles led to changes in bone tissue. This treatment leads to a reduction of bone density of both feet in all used concentrations. But this reduction was statistically significant in a concentration higher than 250 *µ*g/ml. According to these data, it seems that, in a high concentration of Al_2_O_3_ nanoparticles, reduction of bone density is possible. Thus, Al_2_O_3_ nanoparticles have an important role in the change of bone density. Al_2_O_3_ nanoparticles can lead to bone osteoporosis. According to the literature review, no article studies investigated the effect of Al_2_O_3_ nanoparticles on bone density. So, the novelty of this study is that it evaluates the toxicity of Al_2_O_3_ nanoparticles on bone tissue. Other reports have proven the toxicity of different nanostructures on the different organisms (humans, plants, and different other animals) as well as different organs of animals (heart, liver, lung, blood, and brain) [16, 17, 37]. In these reports, it is proven that nanostructures can inhibit, reduce, and defect the activity of organs and so lead to cell death. This study proved that the toxicity of nanostructures on bone tissue could be possible. The behaviors of nanostructures are not similar to normal materials so that these nanostructures can be toxic to cells, tissues, and organs. Agglomeration and aggregation of nanoparticles can change these toxicities. As a result of their size and special properties, nanoparticles sometimes do not behave in the way that normal materials would, and it seems that HA particles can be slightly toxic to cells in the body if they start to clump together, or aggregate. Nanoparticles have higher toxicity at lower concentrations and shorter times in comparison with micro-/macroparticles [38, 39]. Our results showed similar toxicity for Al_2_O_3_ nanoparticles in bone density. Similar to our study, Korani et al. showed that different concentrations of silver nanoparticles had bone toxicities. This group showed that abnormal inflammatory responses and reduction of bone density occurred in nanotreated groups [40]. Based on similar articles, aluminum can accumulate in the bone. This accumulation leads to the occupation of unmineralized type I collagen, destruction of calcification, and resulting in reduction of bone density. Aluminum can impair parathyroid hormone by accumulation in parathyroid glands. Researchers indicated that aluminum displacement of calcium on the bone leads to bone complications such as osteomalacia, hypercalcemia, and hypercalciuria [41–46]. The above suggested mechanisms can be true in the mode of aluminum nanodimensions such as Al_2_O_3_ nanoparticles.

## 5. Conclusion

Based on this study, Al_2_O_3_ nanoparticles can reduce bone density. These results showed the toxicity of these nanostructures on bone tissue. So, due to the increasing use of nanostructures such as Al_2_O_3_ nanoparticles, it must be used by more caution, especially its use as a coating in different devices such as implants, surgical instruments, and bone prostheses.

## Figures and Tables

**Table 1:** Comparison of mean of bone density (g/cm^2^) among all groups (ANOVA test) and control and other groups (Tukey's test).

Groups	Mean ± SD (g/cm^2^)	*p* value	Mean ± SD (g/cm^2^)	*p* value
	Left foot		Right foot	
1	0.1521 ± 0.002		0.1489 ± 0.003	
2	0.1554 ± 0.003	0.988	0.1510 ± 0.003	0.995
3	0.1498 ± 0.001	0.996	0.1485 ± 0.002	0.968
4	0.1387 ± 0.002	0.651	0.1396 ± 0.002	0.689
5	0.1388 ± 0.003	0.668	0.1389 ± 0.002	0.684
6	0.1221 ± 0.001	0.048^*∗*^	0.1232 ± 0.001	0.046^*∗*^
7	0.1159 ± 0.002	0.032^*∗*^	0.1029 ± 0.001	0.016^*∗*^
8	0.1012 ± 0.002	0.021^*∗*^	0.098 ± 0.003	0.009^*∗*^
*p* value	0.036		0.024	

Group definition: 1, control; 2, injection control; 3, treated with 0.5 ml Al_2_O_3_ nanoparticles (25 *µ*g/ml); 4, treated with 0.5 ml Al_2_O_3_ nanoparticles (50 *µ*g/ml); 5, treated with 0.5 ml Al_2_O_3_ nanoparticles (100 *µ*g/ml); 6, treated with 0.5 ml Al_2_O_3_ nanoparticles (250 *µ*g/ml); 7, treated with 0.5 ml Al_2_O_3_ nanoparticles (500 *µ*g/ml); and 8, treated with 0.5 ml Al_2_O_3_ nanoparticles (1000 *µ*g/ml).

## Data Availability

The data used to support the findings of this study are included within the article.
